# Maternal health services utilisation among primigravidas in Uganda: what did the MDGs deliver?

**DOI:** 10.1186/s12992-020-00570-7

**Published:** 2020-05-05

**Authors:** Kilian Nasung Atuoye, Ethel Barnes, Melissa Lee, Lily Ziyue Zhang

**Affiliations:** 1 The Department of Geography, University of Western Ontario, Social Science Centre, 1151 Richmond Street, London, Ontario N6A 5C2 Canada; 2 Department of Schulich, School of Medicine and Dentistry, University of Western Ontario, London, Ontario N6G 5C2 Canada

**Keywords:** Primigravida, Maternal health: antenatal, Skilled birth attendants, SDGs, MDGs, Global Health, Sub-Saharan Africa, Uganda

## Abstract

**Background:**

Achieving maternal health outcomes in the SDGs requires the implementation of more targeted policies and strategies. While the MDGs may have deepened our understanding in this regard, we know little about the trends in maternal health services utilisation among primigravidas, and how age and geographical regions could have influenced these trends. In this study, we examined utilisation of antenatal and skilled delivery services among primigravidas in Uganda, a country with one of the highest maternal mortality ratios, and where early childbearing and its attendant challenges are common.

**Methods:**

Guided by Andersen’s Behavioural Model, we fitted multivariate regression models to a pooled dataset of the 2006, 2011 and 2016 Ugandan Demographic and Health Survey (*n* = 3477) to understand the dynamics in Antenatal Care (ANC) and Skilled Birth Attendance (SBAs) utilisation among primigravidas. Post-estimation margins were employed to further highlight the effect of age and geographical regions.

**Results:**

The analyses show an improvement in access to maternal health services among primigravidas from 2006 to 2016. Compared to 2006, primigravidas in 2016 were 48%, 24% and 2.98 times more likely to have early ANC, four or more ANC visits, and SBAs, respectively. Altogether, a primigravida in 2016 relative to 2006 was 42% more likely to meet all three maternal health service indicators. Post-estimation margins analyses on age and geographical disparities revealed that younger primigravidas have lower probability, while primigravidas in Eastern Region, one of the most deprived in the country, have the lowest probability of accessing maternal health services. Also, the study found education, wealth, women’s household decision-making power, place of residence as important determinants of ANC visits and SBAs.

**Conclusions:**

Based on our findings, it is important to address the vulnerabilities of primigravidas, particularly younger individuals, in accessing early ANC. Uganda should scale-up decentralisation and integration of maternal health delivery in local communities as a strategy of addressing lingering geographical disparities, and ultimately improve maternal health outcomes in the SDGs period.

## Background

The UN Sustainable Development Goal (SDG) 3 prioritizes maternal health, with target 1 working to reduce global Maternal Mortality Ratio (MMR) to less than 70 per 100,000 live births by 2030 [1, 2]. By the end of the MDGs in 2015, significant progress had been made in the area of maternal and child health. Global MMR declined to 216 deaths per 100,000 live births, representing a 37% drop since year 2000, while global under-5 mortality fell to 43 deaths per 1000 live births, a decline of 44% over the same period. Despite this progress, it has been noted that global MMR should decline by about 7.5% annually, more than twice the annual rate of reduction in the MDG period, if the SDG target should be achieved come 2030 [2]. This requires expansion of access to quality maternal health services but also identifying vulnerable groups of women for policy targeting. In this regard, we study the utilisation of maternal health services among first time pregnant women (primigravidas) with the aim of deepening our understanding of and contributing to the conversation around improving maternal and child health in the SDGs period.

The importance of quality maternal health services for mothers and children cannot be overemphasized. Among women with 95% uptake of four or more antenatal visits, in-facilty delivery and Skilled Birth Attendance, MMR is as low as 0–15 per 100,000 live births. In contrast, women with less than 50% uptake of these services report MMR of approximately 500 per 100,000 live births in the MDGs period [3]. This is not suprising because antenatal visits provide an opportunity to identify and address complications during pregnancy, while access to professional services at point of delivery can help manage hemorrhage and postpartum complications which account for about 75–98% of maternal dealths globally [4, 5]. With this understanding, the SDGs have reprioritized increasing antenatal services, assisted, and institutional delivery, while improving nutrition, and strengthening national health systems for effective maternal and fetus assessment, identification of common physiological symptoms, and promotion of preventive healthcare [6].

Access to maternal health services can be constrained by structural, contextual and individual level factors [7–9]. Previous empirical studies have documented the influence of policy in improving access to maternal health services in varied contexts [10–13]. For instance, in rural areas where availability of antenatal care and skilled delivery service is low, women are more likely to miss these services during pregnancy compared to their counterparts in urban localities [14, 15]. Aside availability, socio-economic, socio-cultural, and demographic factors, as well as differences in perception of pregnancy and delivery related risks tend to explain why some women do not utilise maternal services [8, 15].

In Uganda, many births in rural areas take place with the help of traditional birth attendants (TBAs) as a result of inaccessibility of formal health services [16]. For instance, rural localities such as Karamoja, West Nile, Teso, and Bukedi regions have 50 to 60% gap in midwifery staffing which presents significant implications for utilisation of assisted delivery services [17]. Although TBAs are trusted members and have a high social ranking in their local communities, their limited knowledge and lack of formal training have led to risky medical procedures resulting in high rates of complications and even preventable deaths [16]. With renewed impetus following the introduction of the MDGs, the Government of Uganda implemented several policies aimed at improving healthcare access. For example, the country implemented the National Health Sector Strategic Plan (NHSSP I) in 2000, and NHSSP II in 2005 to redirect and retool the countries health sector to improve the delivery of critical health services including maternal and preventive health [11, 18]. Implementation of the Reproductive Health Strategy in 2007 prioritized early pre- and post-natal care, while the National Health Policy II started in 2009 reinforced investment in maternal health and nutrition. In 2015, the country implemented the Uganda Family Planning Costed Implementation Plan to improve allocation of resources to reproductive and other maternal health services [11]. In addition, interventions from non-profit organizations within the MDGs period included the Volunteer Village Health Teams program in 2010 ^19^ and the Labour and Risk Management (ALARM) program in 2015 [19]. Improvements in antenatal visit within first trimester of pregnancy from 17 to 29%, four or more antenatal care visits from 47 to 60%, and skilled birth delivery from 42 to 74% between 2006 and 2016 can be associated with the policies implemented over the MDGs periods [16].

This notwithstanding, there is limited study on disparities in maternal health services utilisation among women with varied delivery experiences. In a socio-cultural context, where pregnancy among unmarried women is stigmatized, and to some extent where pregnant teenagers disproportionately suffer emotional stress, first pregnancy is often hidden away [20–22], and thus reduces primigravidas’ access to maternal health services. The 2016 Ugandan Demographic and Health Survey reports of a growing proportion of women under 20 years in childbearing, majority of whom have earlier start to sexual activities than age at marriage, and record the highest proportion (40%) of mistimed pregnancies. In fact, early (teenage) marriage and ‘informal’ unions – marriage through the window – which is often associated with unplanned pregnancy is on the rise in the country [23]. In addition, approximately 50% of the cost of maternal health services is borne by households in out-of-pocket payments given the low coverage of health insurance (6% in 2016) [16, 24]. Primigravidas would probably report lower access to maternal health services because of the enormous social stigma and financial barrier associated with mistimed pregnancies.

As we journey through the SDGs period, understanding peculiar drawbacks in health access among primigravidas within the context of evolving local ecosocial environment is crucial for more inclusive health policy making. In this regard, we examined two critical questions: 1) how has primigravida’s access to maternal health services changed in the last decade of the MDGs period (2006–2016) in Uganda, and 2) what is the contribution of age (a predisposing factor) and geographical regions (an enabling factor) to these disparities?

## Methods

### Theoretical framework

**Fig. 1:**
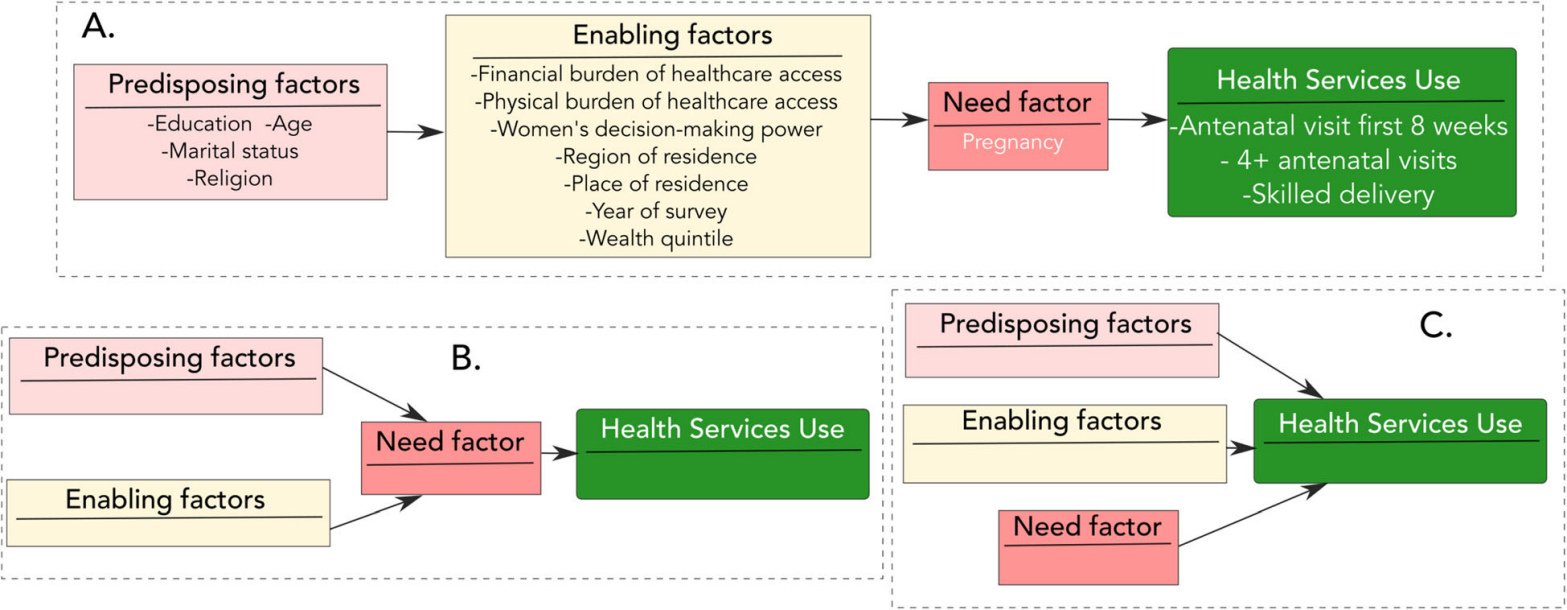
Various configuration of Andersen’s Behavioral Model of Health Service Use (adopted from Graham et al., 2017)

In examining utilisation of maternal health services among primigravidas, we employed Andersen’s behavioural model of health service use as a conceptual guide. Andersen’s model explains the underlying individual and structural level characteristics that promote or impede access to health services [7]. These factors grouped into predisposing, enabling and need factors, interact in multiple ways to influence health service use (see Fig. 1). First, predisposing factors are the sociocultural characteristics that exist prior to a health condition (e.g. educational attainment, social networks, culture/religious beliefs, health beliefs, and demographics).

Second, enabling factors relate to logistical factors that facilitate/help individuals to acquire health services including maternal care (e.g. wealth, social relationships, availability of health care professionals, accessibility of facilities, wait time at point of care, and general health policy context). Third, need factors encompass both perceived and evaluated aspects of health services. They represent an individual’s assessment of the availability and quality of critical services required for addressing a health condition. In maternal health, women who perceive pregnancy as less risky and those who perceive professional services available to them as poor quality are rather more likely to access household and community level traditional maternal health services as found in rural Ghana [14]. This framework has been applied in examining maternal health services utilisation in several context [8, 25–27].

### Data

Data for this study came from a pooled dataset of the 2006, 2011, and 2016 rounds of the Ugandan Demographic and Health Surveys (DHS) collected on women (15–49 years) and men (15–59 years) in a multi-staged sample design, whereby districts are drawn from administrative regions, and classified into Enumeration Areas (EAs) based on type of locality (rural/urban). The DHS program collaborates with the Ugandan Bureau of Statistics (Ugandan government representative) for the data collection. Given its usefulness in health research, DHS data has been applied in the study of health inequalities in several low income countries [25, 26, 28]. We used the women datasets in our analysis.

### Measures

#### Dependent variables: timing and number of ANC visits, SBAs, and all three maternal health services

In tandem with the objective of the study – to understand trends in maternal health services utilisation among primigravidas in the MDGs period, and how age and geographical regions may have influenced these trends – we examined primigravidas’ utilisation of first antenatal visit, the number of antenatal visits, and SBAs. As emphasized by the WHO, these maternal health services are important in ensuring safe pregnancies and deliveries, and therefore contribute to reducing maternal and child mortality as envisaged in SDG 3. The first outcome variable, *timing of first antenatal visit,* was derived from a question on the timing of the first antenatal visit during the last pregnancy and categorized into two (coded: late or never = 0, and early = 1). Early were those who had their first antenatal visit in the first trimester, while late or never were those who had their first antenatal visit after the first trimester of the pregnancy or never had antenatal visit throughout their pregnancy. The second outcome variable, *4+ antenatal visits*, came from a question on total number of antenatal visits during the last pregnancy. This variable was categorized into those who had four or more antenatal visits before delivery and those who did not (coded: less than 4 visits = 0, and at least 4 visits = 1). The third outcome variable, *SBA*, was constructed from a question that asked women the person who assisted them during the delivery of their last pregnancy. This variable was categorized into those who were assisted by a nurse, midwife or a doctor (coded as yes = 1), and those who were not (coded as no = 0), while the final outcome variable combined all the three maternal health variables, categories into primigravidas who met the WHO benchmarks (coded: yes = 1), and those who did not (coded: no = 0).

#### Independent variables

In line with the objective and the conceptual framework guiding the study, we included two blocs of independent variables: predisposing and enabling factors to account for their influence on trends in maternal health services utilisation. The predisposing factors included in our analysis were education, age, marital status, and religion. Enabling factors were household year of survey, wealth quintile, employment status, financial burden of healthcare access, physical burden of healthcare access, women’s decision-making power, place of residence and region of residence. Similar to previous studies [8, 27, 28], pregnancy related health needs served as the need factor in our analysis of antennal and SBAs. Age and region of residence were emphasized as key independent variables given that the study aimed to explain the influence of these two variables on maternal health services utilisation trends. Measurement of these variables are indicated in Table 1.

**Table 1:** Descriptive statistics of maternal health utilisation among primigravidas in Uganda

	Year				Measures and coding of variables
	2006 (*n* = 761)	2011 (*n* = 759)	2016 (*n* = 1957)	2006–16 (*n* = 3477)	
Variable	%	%	%	%	
**Timing of 1st ANC visit**					
≤ 8 weeks - early	22.60	28.72	31.07	28.70	Derived from question on timing of first antenatal visit during last pregnancy. Coded late or never = 0 for ANC after first 8 weeks of pregnancy or no ANC; and early = 1 for first ANC within first 8 weeks.
> 8 weeks - late	77.40	71.28	68.93	71.30	
**ANC visits**					
At least 4 visits	57.56	59.42	64.64	61.95	Came from question on number of antenatal visits during last pregnancy. Coded less than 4 visits = 0, and at least 4 visits = 1.
Less than 4 visits	42.44	40.58	35.36	38.05	
**Skill Birth Attendance**					
Yes	64.65	78.26	87.02	80.21	Person who assisted respondents during last delivery. Coded nurse, midwife or a doctor as yes = 1, and any other as no = 0
No	35.35	21.74	12.98	19.79	
**All maternal health services**					
Yes	13.40	17.79	23.86	20.25	Cross-reference of all three maternal health services. Coded yes = 1 if had early ANC, 4+ ANC visits and assisted delivery, and no = 0 if otherwise
No	86.60	82.21	76.14	79.75	
**Education**					
Primary	61.10	50.99	53.35	54.53	Highest educational attainment of respondents coded Primary = 1, No formal education = 2, and Secondary or higher education = 3
No Form. Edu.	8.28	5.67	3.42	4.98	
Secondary or higher	30.62	43.35	43.23	40.49	
**Age**					
≤ 20	57.82	49.28	49.92	51.51	Age of individuals at time of survey was categorized and coded as ≤20 = 1, 21–25 = 2, and ≥ 26 = 3
21–25	33.51	40.58	38.32	37.76	
26–46	8.67	10.14	11.75	10.73	
**Marital Status**					
Married	78.19	67.72	66.53	69.34	Marital status comprised of never married, widowed, divorced and married, coded into married = 1, and single (never married, widowed and divorced) =2
Single	21.81	32.28	33.47	30.66	
**Religion**					
Christian	85.02	84.45	85.13	84.96	Religion was categorized and coded as Christians = 1, and non-Christians (Moslems, traditional religion and no religion) = 2
Non-Christian	14.98	15.55	14.87	15.04	
**Wealth Quintile**					
Poorest	20.11	17.00	18.96	18.78	Household wealth is a pre-constructed weighted variable in the Ugandan Demographic and Health Survey from household characteristics (e.g. type of house floor materials, wall and roofing), water and sanitation, and electricity availability, household consumption assets (e.g. radio, bicycle, car, etc.).
Poor	19.19	15.68	20.80	19.33	
Middle	15.51	16.34	15.59	15.73	
Rich	14.98	15.68	18.91	17.34	
Richest	30.22	35.31	25.75	28.82	
**Employment status**					
Full	37.32	42.69	45.17	42.91	Employment status came a question about the type of employment of responded, coded into full employment = 1, temporal employment = 2, and unemployed = 3
Temporary	43.63	27.67	32.96	34.14	
Unemployed	19.05	29.64	21.87	22.95	
**Financial access to healthcare**					
Small challenge	38.24	57.97	59.58	54.56	Came from the question on how difficult it is getting money to access healthcare when in need, and this is coded into small challenge = 1 and big challenge = 2
Big challenge	61.76	42.03	40.42	45.44	
**Physical access to healthcare**					
Small challenge	48.49	64.56	66.12	61.92	Came from the question on how difficult it is physically accessing healthcare when in need, and this is coded into small challenge = 1 and big challenge = 2
Big challenge	51.51	35.44	33.88	38.08	
**Women’s decision-making power**					
Middle	13.01	55.60	21.26	55.51	Addictive scale of women’s involvement in decision-making on health care service, large household purchases, visit to relatives, and expending husband’s earnings. Coded as male only (low = 1); men+women (middle = 2), and women only (high = 3). Cronbach’s α: 2006 = 0.82; 2011 = 0.77; and 2016 = 0.79)
Low	86.33	42.95	77.47	43.31	
High	0.66	1.45	1.28	1.18	
**Place of Residence**					
Rural	76.61	60.87	72.87	71.07	Place of residence was coded as rural = 1, and urban = 2
Urban	23.39	39.13	27.13	28.93	
**Region of Residence**					
North	19.32	14.10	15.74	16.16	Derived from a question on district of residence. Political regions were reconfigured in 2011 and 2016. For consistency, regions were reclassified to conform to 2006 categories, and coded North (Karamoja, Lango and Acholi) = 1, Kampala = 2, Central 1 = 3, Central 2 = 4; Eastern Central (Busoga) = 5, Eastern (Bukedi, Bugisu, and Teso) = 6, West Nile = 7, Western (Bunyoro and Tooro) = 8, and Southwestern (Ankole and Kigezi) = 9
Kampala	12.35	16.73	8.84	11.33	
Central 1	9.99	8.30	8.38	8.71	
Central 2	8.67	7.64	7.97	8.05	
East Central	9.86	8.43	6.80	7.82	
Eastern	11.04	11.33	19.57	15.90	
West Nile	9.72	12.91	7.61	9.23	
Western	9.86	9.88	15.07	12.80	
Southwestern	9.20	10.67	10.02	9.98	

### Analytical strategy

**Fig. 2:**
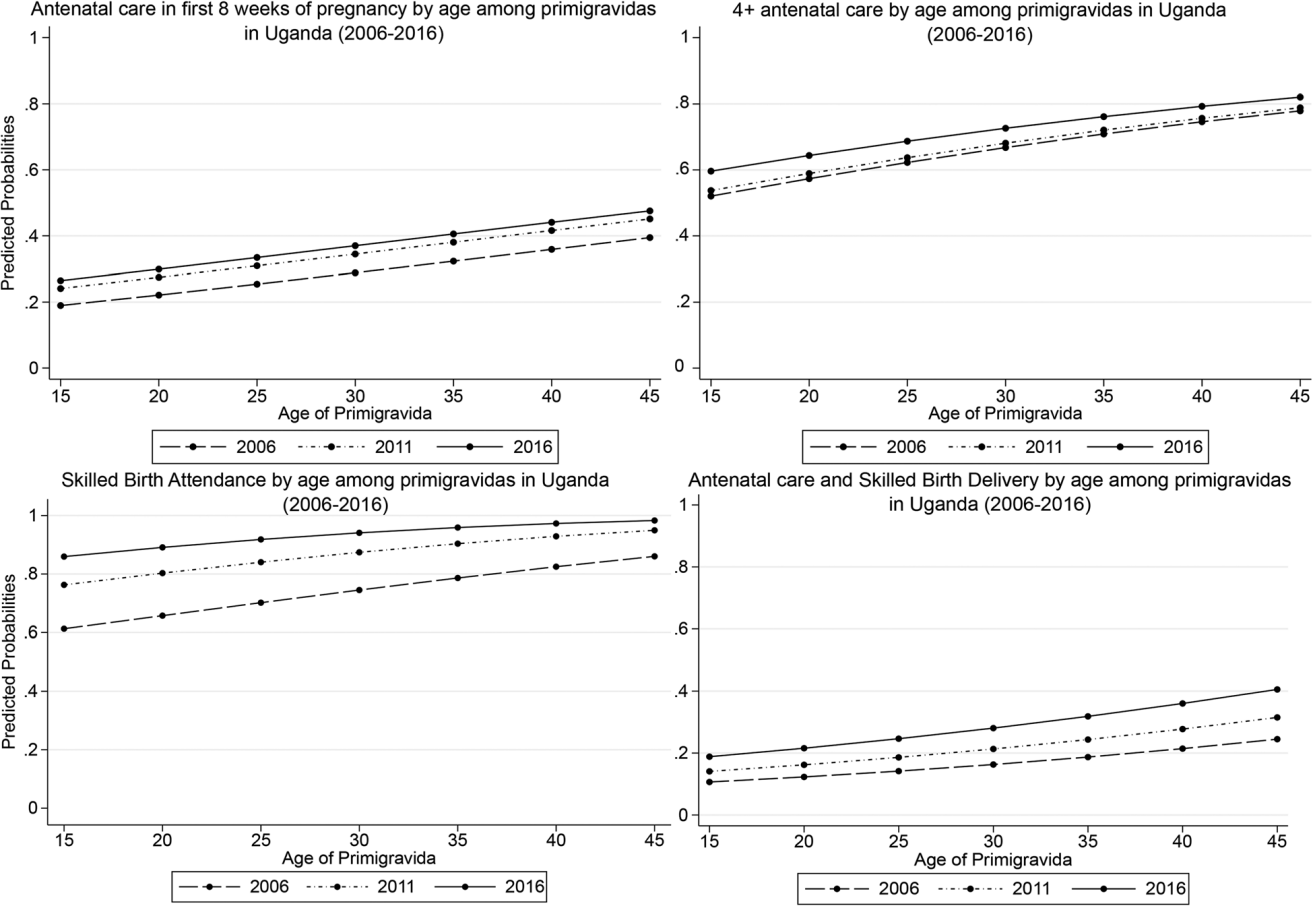
Maternal Health Service Utilisation by age among primigravidas in Uganda (2006–2016)

**Fig. 3:**
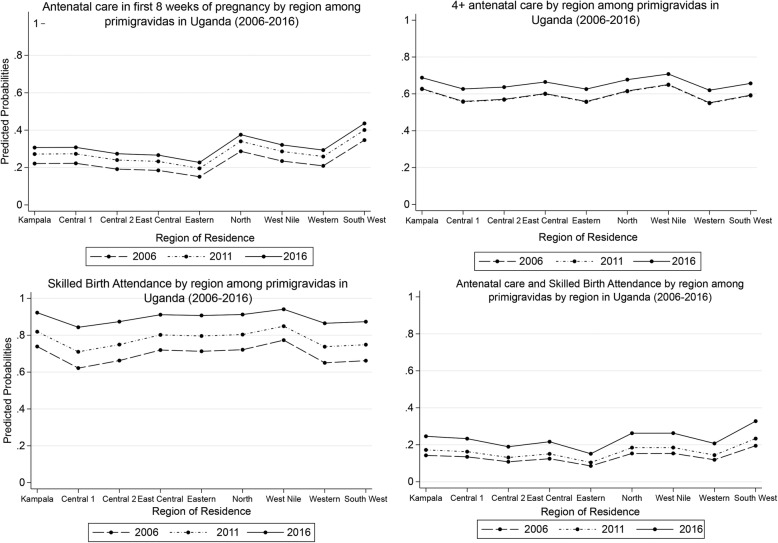
Maternal Health Service Utilization by region among primigravidas in Uganda (2006–2016)

All our four dependent variables were binary and asymmetrically distributed, suggesting that logistic regression which assumes symmetrical distribution in data, would yield biased estimates. For this reason, we employed complementary loglog regressions in the estimation of early ANC, and access to all three maternal health services to normalize the skewedness in the sample distribution (see Table 1). Also, we used negative loglog to estimate primigravidas’ access to four or more ANC visits, and SBAs as these cases of interest were the majority in the sample distribution [29]. These techniques have been deployed in previous studies on maternal health services utilisation in SSA [27, 30]. Additionally, regression models are built with the assumption of independence of responses but as the Ugandan DHS utilised multistage and hierarchical sampling, it is likely that responses from same clusters may be similar. We accounted for the effect of potential clustering by imposing a cluster variable using the vce in STATA. This improved the independence of responses and yielded robust parameter estimates. We built bivariate models to examine initial zero-ordered associations between the four outcomes variables and independent variables (see Supplementary Table 1). Subsequently, four adjusted models were built, one each for timing of first ANC visit, number of the ANC visits, SBA, and access to all three maternal health services (see Table 2). Regression coefficients were exponentiated into Odds Ratios for easy interpretation. Odds ratio > 1 is interpreted as a higher odds, and < 1 is interpreted as a lower odds of utilising a specific maternal health service. We conducted post-estimation margins analyses of the interaction between year of survey and age (see Fig. 2), and between year of survey and region of residence (see Fig. 3) to understand age and geographical disparities in primigravidas’ access to maternal health services utilisation over time. Analyses were limited to only primigravidas, those who had their first pregnancy and delivery in the last 5 years preceding the respective survey, in order to further reduce bias and improve conceptual clarity. This technique reduced the analytical sample from 116,605 to 3477. All analyses were conducted in Stata/SE 15.1.

**Table 2:** Multivariate analysis of ANC and SBAs among primigravidas in Uganda (2006–2016)

	Timing of 1st ANC visit	ANC visits	SBAs	All 3 services
Variable	Adjusted OR(R. SE.)	Adjusted OR(R. SE.)	Adjusted OR(R. SE.)	Adjusted OR(R. SE.)
**Year**				
**2006**	**1.00**	**1.00**	**1.00**	**1.00**
**2011**	1.30 (0.137)**	**0.99 (0.084)**	1.46 (0.154)***	**1.10 (0.074)**
**2016**	1.48 (0.136)***	1.24 (0.092)***	2.98 (0.297)***	1.42 (0.083)***
**Education**				
**Primary**	**1.00**	**1.00**	**1.00**	**1.00**
**No Form. Edu.**	1.54 (0.216)***	**0.90 (0.115)**	0.76 (0.102)**	**1.04 (0.108)**
**Secondary+**	1.15 (0.092)*	1.17 (0.084)**	2.34 (0.291)***	1.16 (0.061)***
**Age**				
**≤ 20**	**1.00**	**1.00**	**1.00**	**1.00**
**21–25**	**1.05 (0.078)**	1.13 (0.073)*	**1.05 (0.092)**	**0.99 (0.049)**
**> 25**	1.33 (0.142)***	1.44 (0.169)***	2.02 (0.443)***	1.25 (0.100)***
**Marital Status**				
**Married**	**1.00**	**1.00**	**1.00**	**1.00**
**Single**	**0.91 (0.107)**	**0.96 (0.087)**	**1.11 (0.140)**	**0.94 (0.071)**
**Religion**				
**Christian**	**1.00**	**1.00**	**1.00**	**1.00**
**Non-Christian**	**1.12 (0.104)**	**1.03 (0.085)**	**0.97 (0.119)**	**1.11 (0.070)**
**Wealth Quintile**				
**Richest**	**1.00**	**1.00**	**1.00**	**1.00**
**Poorest**	0.77 (0.106)*	0.67 (0.087)***	0.43 (0.083)***	0.81 (0.077)**
**Poor**	0.76 (0.097)**	0.65 (0.078)***	0.48 (0.090)***	0.80 (0.069)***
**Middle**	**0.82 (0.105)**	0.76 (0.090)**	0.59 (0.112)***	0.84 (0.072)**
**Rich**	**0.86 (0.098)**	0.73 (0.079)***	0.65 (0.118)**	**0.89 (0.070)**
**Employment**				
**Full**	**1.00**	**1.00**	**1.00**	**1.00**
**Temporary**	**0.88 (0.069)**	**0.93 (0.064)**	**0.96 (0.090)**	0.90 (0.048)*
**Unemployed**	**0.97 (0.081)**	0.88 (0.065)*	**1.18 (0.140)**	**0.96 (0.055)**
**Financial burden of healthcare access**				
**Small challenge**	**1.00**	**1.00**	**1.00**	**1.00**
**Big challenge**	**0.89 (0.064)**	**0.96 (0.060)**	1.19 (0.105)*	**0.98 (0.048)**
**Physical burden of healthcare access**				
**Small problem**	**1.00**	**1.00**	**1.00**	**1.00**
**Big problem**	**0.996 (0.074)**	**0.91 (0.057)**	0.83 (0.070)**	**0.96 (0.048)**
**Women’s decision-making power**				
**Middle**	**1.00**	**1.00**	**1.00**	**1.00**
**High**	**1.04 (0.287)**	**0.82 (0.205)**	0.63 (0.173)*	**0.97 (0.195)**
**Low**	**0.93 (0.101)**	0.81 (0.069)**	**0.84 (0.094)**	**0.94 (0.066)**
**Place of Residence**				
**Rural**	**1.00**	**1.00**	**1.00**	**1.00**
**Urban**	0.82 (0.080)**	0.86 (0.077)*	1.91 (0.321)***	**0.94 (0.064)**
**Region of Residence**				
**North**	**1.00**	**1.00**	**1.00**	**1.00**
**Kampala**	0.76 (0.115)*	**1.04 (0.155)**	**1.39 (0.455)**	**0.99 (0.105)**
**Central 1**	0.76 (0.112)*	**0.84 (0.112)**	**0.83 (0.158)**	**0.95 (0.098)**
**Central 2**	0.65 (0.098)***	**0.86 (0.111)**	**0.79 (0.135)**	0.81 (0.081)**
**East Central**	0.63 (0.098)***	**0.95 (0.125)**	**1.19 (0.217)**	**0.92 (0.093)**
**Eastern**	0.52 (0.066)***	0.83 (0.084)*	**1.04 (0.135)**	0.73 (0.059)***
**West Nile**	0.80 (0.109)*	**1.13 (0.136)**	1.33 (0.199)*	**1.02 (0.094)**
**Western**	0.72 (0.090)***	0.81 (0.090)*	**0.85 (0.122)**	0.86 (0.075)*
**Southwestern**	1.24 (0.149)*	**0.93 (0.114)**	**0.83 (0.124)**	1.17 (0.108)*
**Constant**	0.41 (0.069)***	3.00 (0.460)***	8.86 (1.999)***	0.63 (0.073)***
**Observation**	**3477**	**3477**	**3477**	**3477**

Note:*OR*Odds ratios,*R. SE*. robust standard errors in parenthesis; ***p < 0.01, **p < 0.05, *p **< 0.1**

## Results

We present the sample characteristics highlighting the relative changes in utilisation of antenatal and skilled birth deliveries service among primigravidas in the MDG period in Uganda. We also present the independent effect of policy changes (captured in year of survey), predisposing factors, and enabling factors, and then conclude the section with a closure look at the changing influence of age and geographical disparities in maternal health services utilisation in 2006, 2011 and 2016.

### Sample characteristics

The sample characteristics are presented in Table 1. The study found marginal improvement in early ANC utilisation. For instance, while about 23% had ANC in the first 8 weeks of pregnancy in 2006, about 29 and 31% achieved this target in 2011 and 2016, respectively, with an average of 29% having early ANC in the last 10 years of the MDGs. Also, the proportion of primigravidas who had 4+ ANC visits increased slightly from 58% in 2006 to 59% in 2011, and further to 65% in 2016. On the average, 62% of primigravidas utilised 4+ ANC visits from 2006 to 2016. Access to SBAs however improved remarkably. In 2006, 65% of primigravidas accessed SBAs, but this jumped to 87% in 2016 after rising to 78% in 2011.

Analysis of the pooled data shows that 80% of primigravidas accessed SBAs between 2006 and 2016. These findings are similar to the maternal health services utilisation trends in the country [16], except that primigravidas’ access to ANC is lower while their utilisation of SBAs is higher than the general population. The study showed heterogeneities in predisposing and enabling factors to healthcare use among primigravidas as shown in Table 1.

### Early antenatal care access

For brevity and to focus the paper on independent effects, we report only adjusted results from multivariate regression presented in Table 2. However, results from bivariate analysis are reported in Supplementary Table 1. The multivariate analyses show that primigravidas in 2011 were 30% more likely, and in 2016, 48% more likely to utilise early ANC compared to 2006. Predisposing factors such as educational attainment and age were associated with disparities in the utilisation of early antenatal services. Primigravidas with no formal education and those with secondary education or higher compared with their colleague with primary education were more likely to have early ANC (AOR = 1.54, *p* ≤ 0.001; and AOR = 1.15, *p* ≤ 0.1, respectively). Similarly, primigravidas above 25 years were more likely to utilise early ANC (AOR = 1.33, p ≤ 0.001) compared to those younger than 21 years. For enabling factors, household wealth and locational characteristics were associated with early ANC utilisation. In contrast with individuals from the richest households, those from the poorest and poor households were less likely to have early ANC (AOR = 0.77, p ≤ 0.1; and AOR = 0.76, *p* ≤ 0.05, respectively). Surprisingly, primigravidas in urban settings were about 18% less likely to utilise early ANC compared to their counterparts in rural areas. Further analysis (not reported here) revealed wealth disparities as explaining this seemingly counter-intuitive finding. Also, aside Southwestern region, which showed higher odds, all other regions reported lower odds of early ANC utilisation relative to North region.

### Meeting 4+ antenatal care

Primigravidas were 24% more likely to access four or more ANC visits in 2016 than in 2006, with no statistically significant difference between 2011 and 2006. Consistent with early ANC, disparities in utilisation of 4+ ANC visits were associated with education and age. Primigravidas with secondary education were more likely to have 4+ ANC visits (AOR = 1.17, *p* ≤ 0.05) compared with those with primary education, while those aged 21–25 and 25 years or above were more likely to report higher odds of utilising 4+ ANC visits (AOR = 1.13, *p* ≤ 0.1; and AOR = 1.44, *p* ≤ 0.001, respectively) relative to younger primigravidas (below 20 years). However, unlike early ANC, other enabling factors in addition to wealth and locational factors showed statistically significant association with 4+ ANC visits. For example, primigravidas who were unemployed were associated with lower odds of having 4+ ANC visits (AOR = 0.88, *p* ≤ 0.1) compared with their counterparts in full employment. Similarly, primigravidas with lower level of household decision-making power (involves both males and females) were 19% less likely to utilise 4+ ANC visits relative to those with middle level (males only). Furthermore, primigravidas in rich (AOR = 0.73, *p* ≤ 0.001), middle (AOR = 0.76, *p* ≤ 0.05), poor (AOR = 0.65, *p* ≤ 0.001) and poorest (AOR = 0.67, *p* ≤ 0.001) households were less likely to access 4+ ANC visits compared to those in richest households. Just as in early ANC, urban compared to rural residence was associated with lower odds of utilising 4+ ANC visits (AOR = 0.86, *p* ≤ 0.1), while Eastern and Western regions compared to North region were less likely to utilise 4+ ANC visits (AOR = 0.83, *p* ≤ 0.1, and AOR = 0.81, *p* ≤ 0.1, respectively).

### Skilled birth attendance (SBAs) utilisation

Access to SBA showed the highest improvement in the decade to the end of the MDG period in the study. Compared to 2006, primigravidas were 46% more likely in 2011, and about 3 times more likely to utilise skilled delivery services in 2016. Again, education and age were the only statistically significant predisposing factors predicting access to SBAs. While primigravidas with secondary education or higher were more likely (AOR = 2.34, *p* ≤ 0.001), those with no formal education were less likely to have SBAs (AOR = 0.76, p ≤ 0.001) compared to their counterparts with primary education. Similarly, older primigravidas (> 25 years) were more than 2 times more likely to utilise SBAs relative to younger primigravidas (≤20 years). The influence of enabling factors was particularly more pronounced in SBAs than in antenatal care. Here, financial and physical burden of health care access, which were less important in antenatal care use, gain prominence in skilled delivery service utilisation. Primigravidas with huge physical challenge in accessing healthcare were less likely to utilise SBAs (AOR = 0.83, *p* ≤ 0.05) than those with less physical challenge. However, financial burden being a huge challenge to healthcare was rather associated with higher likelihood of utilising SBAs (AOR = 1.19, *p* ≤ 0.1). In addition, primigravidas with higher level of decision-making power were less likely to use skilled delivery services than those with middle level of decision-making power (AOR = 0.63, *p* ≤ 0.1). Wealth and geographical disparities also influence access to SBAs in this study.

### Counting progress against the WHO recommended maternal health indicators

The standard of measuring progress in the MDG period was the WHO recommendation that every woman should utilise antenatal care service in the first trimester of pregnancy, have at least 4 antenatal visits before delivery and utilise skilled delivery services during delivery. Taken together, primigravidas were 42% more likely to meet the WHO recommendation in 2016 compared to 2006. Nonetheless, disparities were found along predisposing (i.e. education and age), and enabling factors (i.e. household wealth, employment status and region of residence). Higher educational attainment (secondary+) compared to primary education, and primigravidas above 25 years compared to those aged ≤20 were more likely to meet the WHO recommended maternal health services utilisation (AOR = 1.16, *p* ≤ 0.001; and AOR = 1.25, *p* ≤ 0.001, respectively). In addition, Primigravidas in poorest, poorer, and middle wealth households were less likely to meet the WHO recommendation compared to those in the richest households (AOR = 0.81, *p* ≤ 0.05; AOR = 0.80, *p* ≤ 0.001; and AOR = 0.84, *p* ≤ 0.05, respectively). Individuals in temporary employment where less likely to meet the WHO recommended maternal health services utilisation (AOR = 0.90, *p* ≤ 0.1) than those in full employment. Similarly, while primigravidas in Central 2, Eastern and Western regions were less likely, those in Southwestern were more likely to meet the WHO recommended maternal health services utilisation service level compared to their counterparts in North region (AOR = 0.81, *p* ≤ 0.05; AOR = 0.73, *p* ≤ 0.001; AOR = 0.86, *p* ≤ 0.1, and AOR = 1.17, *p* ≤ 0.1, respectively). Surprisingly, rural-urban inequalities were less important in the attainment of the WHO recommended maternal health services utilisation.

### Closer look at age and geographical disparities in antenatal care and SBAs use over time

As indicated in Fig. 2, the study found that much of the improvement in access to early antenatal visits and skilled birth delivery was between 2006 and 2011, but for 4+ antenatal visits and meeting the three WHO recommended maternal health indicators, a significantly greater improvement was seen between 2011 and 2016.

Age disparities was associated with access to maternal health services. For instance, a primigravida aged 45 was about two times more likely to access early ANC and about three times more likely to meet all three WHO recommended maternal health services compared to their counterpart aged 15 in 2016. Among all the maternal health indicators, the greatest improvement was reported in access to SBAs – a change in probability from 0.61 for 15-year old primigravida in 2006 to 0.98 for 45-year old primigravida in 2016. In contrast, the lowest improvement was reported in access to all the WHO recommended maternal health services with a change in probability from 0.11 for primigravidas aged 15 in 2006 to 0.41 for those aged 45 in 2016.

Similar dynamics were revealed across geographical regions over the three-time periods (see Fig. 3). Overall, the lowest probability of accessing early ANC and the WHO recommended maternal health services was reported in Eastern region. Central 1 and Western had the lowest probability in utilisation 4+ ANC and SBAs, respectively.

In contrast, the highest probabilities of accessing early ANC visit and all the WHO recommended maternal health services were reported in South West region, and for 4+ ANC and SBAs in West Nile region.

## Discussion

In this study, we examined trends in maternal health services utilisation among primigravidas in the last decade of the MDGs period (2006 to 2016) in Uganda, and how age and geographical location influenced these trends. Overall, the study found that maternal health services utilisation among primigravidas improved over the study period, with noticeable age and location-based disparities. These findings largely reflect the commitment of the government of Uganda and other actors in the health sector to improve coverage and quality of healthcare during the MDG period. Significant investments were made to train and deploy health professionals, establish health facilities, improve medical equipment supplies, decentralize health care, and implement maternal health education programs [11, 18, 19, 31]. The SDGs present an opportunity to further improve maternal health services for diverse groups of women in the context of guaranteeing reproductive health rights, an apparent omission in the MDGs [32]. In this regard, the findings here are important in not only explaining dynamics in maternal health services utilisation among a unique group of women, but they also provide evidence for advancing reproductive health rights through health policymaking in Uganda and similar context.

Despite reporting relatively higher utilisation of maternal health services than the general women population [11, 16], primigravidas had a smaller improvement in access to these services in the MDGs period. For instance, while antenatal visit in first trimester of pregnancy, 4+ antenatal visits, and access to skilled delivery increased by 71, 28 and 76%, respectively between 2006 and 2016 among the general women population, the corresponding change among primigravidas was 37, 12, and 35%, respectively (see Table 1). It is likely the burden of socio-cultural barriers to health access is far greater for primigravidas, and policies implemented during the MDGs period probably made more impact on other categories of women than primigravidas. Generally, pregnant women may miss the timing of first ANC visit because of poor knowledge of noticing pregnancy at the early stage or when to commence antenatal visits [33, 34]. Also, women with mistimed/unplanned or unwanted pregnancy tend to delay in commencing antenatal visits as they hope for the disappearance of the pregnancy [22, 35, 36]. For primigravidas, these dynamics are even more pronounced. In the context of Uganda, a significant number of primigravidas are teenagers and unmarried [23, 37]. Unlike older primigravidas, this group of women are more likely to miss early ANC (see Fig. 2) because of stigmatization from community, fear of punishment/reprehension from relatives for indulging in early sex [37, 38], coupled with their weak position in health seeking decision making in households [39, 40]. Given these findings, we join previous works in emphasizing the need for greater efforts at reducing stigma and socio-economic barriers impeding primigravidas’ access to healthcare, while at the same time advocating for empowerment of young women’s in health seeking choices. These strategies could go a long way to improve primigravidas’ access to maternal health services [23, 40].

Nevertheless, it is possible that work by NGOs on girls’ education, reproductive health, and the Uganda’s maternal health programs are having a greater positive impact on primigravida’s access to skilled birth delivery than antenatal services as shown in the study. This is not surprising because the emphasis of most maternal health promotion activities center on reducing the country’s high mortality and morbidity level. With the burden of financial and physical cost to healthcare access, many women prioritize accessing professional care at point of delivery which is considered most critical for averting chronic complications and deaths [31]. In addition, first delivery anxiety may be drawing primigravidas to professional care at delivery. This finding is consistent with previous studies that highlight the influence of delivery experience in disparities in maternal health services utilisation even in contexts where maternal health services are free [8, 9, 12, 34]. The SGDs present an opportunity to increase access to antenatal services by implementing policies and programs that reduce health access burden and empowers young primigravidas to initiate early access to healthcare.

However, locational disparities present a unique challenge for access to maternal health services. The study found that primigravidas in rural settings where the gap in health personnel staffing position is relatively high were less likely to utilise SBA, but more likely to access antenatal services. Even though community health workers are promoting maternal health in rural communities, primigravidas may not have immediate access to professional health services at time of delivery. Indeed, the study found primigravidas with a huge burden of physical access to healthcare to have low utilisation of SBAs. Furthermore, regional disparities show that primigravidas in Eastern region had the lowest access to early antenatal and the three maternal health services indicators combined, while those in Western and Central 1 reported the lowest access to 4+ antenatal visits and skilled deliveries, respectively (see Fig. 3). These regions are among the most deprived in Uganda. For instance, Eastern region (comprising Bukedi, Teso, and Bugisu) has relatively higher fertility rate (ranging between 5.6 in Bugisu and 6.1 in Bukedi versus 5.4 national average), higher poverty incidence (50 to 90% of population in poorest and poor household wealth quintiles) [16], and also 50 to 60% gap in midwifery staffing [17]. In these regions, Traditional Birth Attendants continue to play a crucial role in deliveries as a temporary measure [16].

Although peripheral, the study found other predisposing and enabling factors important in maternal health services utilisation. For instance, primigravidas who feel the cost of healthcare is a major burden would prioritise investment in SBA services [8]. In addition, while higher educational attainment acts as catalyst for maternal health services utilisation, the difference between primary and no education seems more complex. It is possible community health education programs implemented by NGOs and Village Health Volunteer Teams have a greater positive impact on primigravidas with no formal education than those with primary education in access to antenatal services, but not in SBA which often involves a greater cost. Aside these nuances, the influence of education on maternal health services utilisation is well documented in previous studies [15, 26]. Furthermore, the participation of both men and women in household decision-making is associated with primigravidas’ access to maternal health services – particularly skilled delivery and 4+ antennal visits. In the context of Uganda, households are organized in extended families where the head and elderly women in the household play a critical role in household decision-making including access to maternal health care [23]. This makes the participation of adult men (e.g. household heads) and mothers in-law in maternal health sensitization/education critical in increasing primigravidas’ access to important health services in the SGDs period in Uganda as demonstrated in other context [41].

Consistent with the literature [8, 15, 25, 33, 42], household wealth provided an impetus for utilisation of antenatal visits and assisted delivery by primigravidas in Uganda. From an economic viewpoint, wealthier households can provide resources for more timely access to maternal health services than poorer household. They are also able to afford and access media tools such as TV, radio and newspapers, often employed to inform the Ugandan population about health policies and programs [16]. Through this, primigravidas in wealthier households are more empowered to utilise maternal health services. Surprisingly, the impact of wealth disparities was minimal for the timing of first ANC visit probably because of primigravidas’ inexperience and poor knowledge on signs of pregnancy and when to initiate first ANC. This is consistent with studies in Kampala [34] and in Ghana [33] which showed less disparities in early antenatal visit. Relatedly, primigravidas in full employment may have job-related support for health access, and are therefore more likely to utilise maternal health services [11, 31].

We note some limitations with this study. First, given that the study was cross-sectional, the findings should be interpreted as associations. Second, there is the potential for recall bias as the reference was 5 years preceding the data collection. Thirdly, social desirability among respondents may bias the responses as pregnancy outside marriage is stigmatized in the context of Uganda. Lastly, other factors relevant in maternal health services utilisation such availability of health facilities and personnel were not part of the data, and therefore we employed perception of difficulty in financially and physically accessing health facilities as proxies. Nonetheless, our findings are consistent with the literature and provide important imperatives for maternal health policy in Uganda and similar developing countries in the SDGs period.

## Conclusion

Uganda has made great strides on maternal health in the last decade. However, of particular concern is the fact that commencement of antenatal visits among primigravidas is improving at a relatively slower pace than other maternal health services. Additionally, younger primigravidas and those in the Eastern Region of the country were less likely to utilise maternal health services, confirming the lingering effect of predisposing and enabling factors in maternal health disparities. While these inequalities may not lead to a general decline in maternal health services use among primigravidas in Uganda, there is the need to re-assess policies aimed at promoting healthy motherhood and reproductive health rights in the context of universal health coverage. We recommend further decentralisation of maternal health services to local communities. For instance, community health personnel including traditional birth attendants should be trained on provision of basic antenatal service provision, early detection of pregnancy complications and implementation of effective referral system to support skilled delivery. Innovative community level referral system in which community members contribute a fund in support of referral and transportation of women in labour to delivery points should be implemented. Overall, the findings in this study support an urgent need for a targeted health policy that would reduce the burden of lingering socio-economic barriers to maternal health care access and advance reproductive health rights in Uganda and other low-income countries in the SDGs period.

## Supplementary information

**Additional file 1: Table S1.** Bivariate analysis of ANC and SBAs services utilisation among primigravidas in Uganda (2006-2016).

## Data Availability

Data and materials used in the study are available online on the Demographic and Health Survey website (see link: https://dhsprogram.com/Data/).

## Abbreviations

MDGs: Millennium Development Goals
SDGs: Sustainable Development Goals
MMR: Maternal Mortality Ratio
SSA: Sub-Saharan Africa
WHO: World Health Organization
UN: United Nations
UHC: Universal health Coverage
ANC: Antenatal Care
UNFPA: United Nations population Fund (formerly: United Nations Fund for Population Activities)
TBAs: Traditional Birth Attendants
UBOS: Uganda Bureau of Statistics
NHSSP: National Health Sector Strategic Plan
DHS: Demographic and Health Survey
USAID: United States Agency for International Development
EAs: Enumeration Areas
SBA: Skill Birth Attendants
OR: Odds Ratio
UDHS: Ugandan Demographic and Health Survey
NISR: National Institute of Statistics of Rwanda
