# Supplementary material for: Maternal health services utilisation among primigravidas in Uganda: what did the MDGs deliver?
Source: Global Health. 2020 May 5;16:40. doi: 10.1186/s12992-020-00570-7 (PMC7201536; doi:10.1186/s12992-020-00570-7)
Supplement: Supplementary file 1 — Additional file 1: Table S1. Bivariate analysis of ANC and SBAs services utilisation among primigravidas in Uganda (2006-2016). [file 12992_2020_570_MOESM1_ESM.docx]

**Supplementary Table 1:** Bivariate analysis of ANC and SBAs among primigravidas in Uganda (2006-2016)

|  | **Timing of 1^st^ ANC visit** | **ANC visits** | **SBAs** | **All 3 services** | |  |
| --- | --- | --- | --- | --- | --- | --- |
| **Variable** | **Unadjusted**  **OR(R. SE.)** | **Unadjusted**  **OR(R. SE.)** | **Unadjusted**  **OR(R. SE.)** | | **Unadjusted**  **OR(R. SE.)** | |
| **Year** |  |  |  | |  | |
| 2006 | 1.00 | 1.00 | 1.00 | | 1.00 | |
| 2011 | 1.32(0.135)*** | 1.06(0.086) | 1.78(0.177)*** | | 1.36(0.179)** | |
| 2016 | 1.45(0.126)*** | 1.27(0.086)*** | 3.14(0.276)*** | | 1.89(0.207)*** | |
| **Education** | |  |  | |  | |
| Primary | 1.00 | 1.00 | 1.00 | | 1.00 | |
| No Form. Edu. | 1.64(0.217)*** | 0.90(0.108) | 0.60(0.077)*** | | 1.07(0.205) | |
| Secondary+ | 1.31(0.086)*** | 1.46(0.087)*** | 4.22(0.457)*** | | 1.69(0.131)*** | |
| **Age** |  |  |  | |  | |
| ≤20 | 1.00 | 1.00 | 1.00 | | 1.00 | |
| 21-25 | 1.18(0.081)** | 1.29(0.077)*** | 1.59(0.132)*** | | 1.25(0.104)*** | |
| 26-46 | 1.66(0.159)*** | 1.90(0.208)*** | 4.32(0.905)*** | | 2.14(0.229)*** | |
| **Marital Status** |  |  |  | |  | |
| Married | 1.00 | 1.00 | 1.00 | | 1.00 | |
| Single | 0.86(0.061)** | 0.85(0.050)*** | 1.33(0.117)*** | | 0.88(0.074) | |
| **Religion** |  |  |  | |  | |
| Christian | 1.00 | 1.00 | 1.00 | | 1.00 | |
| Non-Christian | 1.04(0.091) | 1.09(0.086) | 1.232(0.141)* | | 1.20(0.120)* | |
| **Wealth Quintile** |  |  |  | |  | |
| Richest | 1.00 | 1.00 | 1.00 | | 1.00 | |
| Poorest | 0.80(0.075)** | 0.60(0.050)*** | 0.17(0.024)*** | | 0.53(0.062)*** | |
| Poor | 0.75(0.071)*** | 0.61(0.051)*** | 0.20(0.030)*** | | 0.55(0.063)*** | |
| Middle | 0.83(0.082)* | 0.67(0.060)*** | 0.24(0.038)*** | | 0.63(0.074)*** | |
| Rich | 0.87(0.082) | 0.69(0.061)*** | 0.34(0.055)*** | | 0.72(0.081)*** | |
| **Employment** |  |  |  | |  | |
| Full | 1.00 | 1.00 | 1.00 | | 1.00 | |
| Temporary | 0.81(0.060)*** | 0.79(0.050)*** | 0.61(0.051)*** | | 0.64(0.058)*** | |
| Unemployed | 0.90(0.073) | 0.85(0.061)** | 1.24(0.140)* | | 0.86(0.082) | |
| **Financial burden of healthcare access** | |  |  | |  | |
| Small challenge | 1.00 | 1.00 | 1.00 | | 1.00 | |
| Big challenge | 0.82(0.053)*** | 0.80(0.045)*** | 0.68(0.052)*** | | 0.74(0.057)*** | |
| **Physical burden of healthcare access** | |  |  | |  | |
| Small **challenge** | 1.00 | 1.00 | 1.00 | | 1.00 | |
| Big **challenge** | 0.88(0.059)* | 0.77(0.043)*** | 0.51(0.039)*** | | 0.72(0.059)*** | |
| **Women’s decision-making power** | |  |  | |  | |
| Middle | 1.00 | 1.00 | 1.00 | | 1.00 | |
| High | 1.13(0.309) | 0.74(0.179) | 0.51(0.141)** | | 0.87(0.310) | |
| Low | 0.81(0.053)*** | 0.78(0.044)*** | 0.96(0.075) | | 0.78(0.061)*** | |
| **Place of Residence** | |  |  | |  | |
| Rural | 1.00 | 1.00 | 1.00 | | 1.00 | |
| Urban | 1.10(0.076) | 1.32(0.085)*** | 4.77(0.645)*** | | 1.47(0.115)*** | |
| **Region of Residence** | |  |  | |  | |
| North | 1.00 | 1.00 | 1.00 | | 1.00 | |
| Kampala | 0.94(0.111) | 1.59(0.186)*** | 8.44(2.282)*** | | 1.55(0.212)*** | |
| Central 1 | 0.94(0.120) | 1.15(0.135) | 1.82(0.296)*** | | 1.34(0.204)* | |
| Central 2 | 0.73(0.103)** | 1.04(0.122) | 1.41(0.218)** | | 0.90(0.157) | |
| East Central | 0.69(0.101)** | 1.09(0.131) | 1.61(0.263)*** | | 0.99(0.170) | |
| Eastern | 0.55(0.067)*** | 0.93(0.087) | 1.43(0.177)*** | | 0.64(0.099)*** | |
| West Nile | 0.80(0.105)* | 1.17(0.135) | 1.40(0.205)** | | 1.05(0.167) | |
| Western | 0.81(0.096)* | 0.96(0.096) | 1.32(0.170)** | | 0.97(0.144) | |
| Southwestern | 1.40(0.156)*** | 1.18(0.133) | 1.25(0.172) | | 1.67(0.232)*** | |
| **Observation** | 3,477 | 3,477 | 3,477 | | 3,477 | |

Note: OR=Odds ratios; R. SE.=robust standard errors in parenthesis; *** p<0.01, ** p<0.05, * p<0.1
